# Co-occurring Agamid Lizards Vary in Thermal Vulnerability Despite Similar Climatic Exposure

**DOI:** 10.1093/iob/obag041

**Published:** 2026-07-24

**Authors:** A Ben, M Razak, M Thaker

**Affiliations:** Centre for Ecological Sciences, Indian Institute of Science, Bangalore 560012, India; Centre for Ecological Sciences, Indian Institute of Science, Bangalore 560012, India; Indian Institute of Science Education and Research, Tirupati 517619, India; Centre for Ecological Sciences, Indian Institute of Science, Bangalore 560012, India

## Abstract

Global warming increasingly exposes ectotherms to temperatures near or beyond their physiological limits, threatening survival and persistence. Vulnerability of species to rising temperatures is shaped by multiple factors, including the thermal environments they experience, their physiological tolerances, and the temperature dependence of key performance traits. Here, we investigated the thermal ecology and climate vulnerability of two co-occurring tropical agamid lizards from semi-arid Southern India: the arboreal generalist *Calotes versicolor* and the saxicolous specialist *Psammophilus dorsalis*. For both species, we measured microenvironmental temperatures using physical copper models in open and shaded microhabitats, and quantified field-active body temperatures (Tb), as well as measured preferred temperature range (Tpref), critical thermal limits (CTmin and CTmax), and determined thermal performance curves for sprint speed and bite force. Thermal vulnerability indices (thermoregulatory accuracy, habitat thermal quality based on physical models, effectiveness of thermoregulation, warming tolerance, and thermal safety margins) were calculated and we projected future performance declines under IPCC Shared Socioeconomic Pathways (SSP1-2.6, SSP2-4.5, and SSP5-8.5). Although both species experienced similar microhabitat temperatures, they differed in thermoregulatory strategies and vulnerability. The widely distributed *C. versicolor* had a wider Tpref range, higher CTmax, lower CTmin, and broader thermal tolerance range. This species also had greater thermoregulatory effectiveness in open microhabitats, lower deviations from preferred temperatures, and more favorable habitat thermal quality. In contrast, *P. dorsalis* displayed narrower Tpref range, lower CTmax, higher CTmin, narrower thermal tolerance, and reduced thermoregulatory accuracy and effectiveness. As a consequence, *P. dorsalis* was operating closer to its upper thermal limits and experiencing reduced warming tolerance. Sprint speed had higher thermal optima and narrower performance breadths than bite force in both species, rendering locomotion more sensitive to warming. Projections indicated minimal impacts on both sprint speed and bite force, even under high-emission scenarios. Nevertheless, for the saxicolous *P. dorsalis*, narrow warming tolerances and thermal safety margins, suggest potential vulnerability through other ecological and physiological axes. Our findings reveal that closely related co-occurring lizards can occupy different thermal niches and face unequal climate risks despite shared ambient conditions. Species differences in microhabitat specialization, rather than exposure alone, emerges as a key driver of thermal vulnerability, underscoring its critical role in shaping resilience to ongoing climate warming.

## Introduction

Climate change is reshaping species distributions, phenology, and extinction risk worldwide (Parmesan and Yohe 2003; Sinervo et al. 2010). Ectotherms are particularly vulnerable because their core physiological processes, such as metabolism, digestion, locomotion, and reproduction are tightly coupled to environmental temperature. When ambient conditions exceed physiological thresholds, survival and persistence become constrained (Deutsch et al. 2008; Angilletta Jr. 2009; Huey et al. 2012). Thermal sensitivity is especially pronounced in the tropics, where species have evolved under relatively stable thermal regimes and often operate close to their upper thermal limits with narrow safety margins (Deutsch et al. 2008; Tewksbury et al. 2008; Kingsolver et al. 2013). Accurate assessment of warming vulnerability therefore requires integrating climate projections with species-specific thermal tolerances and performance capacities (Sunday et al. 2014).

Thermal physiology sets fundamental bounds on organismal function, defining the temperature range over which key performance traits remain effective (Huey and Stevenson 1979; Angilletta Jr. 2009). In the wild, ectotherms vary widely in their thermoregulatory strategies to achieve suitable body temperatures. Species can range from being thermoconformers, whose body temperatures closely track ambient conditions, to thermoregulators, which actively maintain optimal body temperatures through physiological and behavioral adaptations, such as basking, microhabitat selection, and postural adjustments (Huey and Slatkin 1976; Stevenson 1985; Angilletta Jr. 2009). The use of these strategies depends on both intrinsic physiological traits and the thermal environments available to individuals. Spatial heterogeneity in microclimates enables ectotherms to behaviorally buffer environmental variation and maintain body temperatures that optimize performance, thereby influencing realized thermal niches (Angilletta Jr. 2009; Pincebourde et al. 2016).

Among closely related species, upper thermal limits often exhibit strong phylogenetic conservatism, reflecting shared evolutionary history and similarities in thermal ecology (Sunday et al. 2011; Gunderson et al. 2018). However, experimental and quantitative genetic studies indicate that both CTmax and CTmin are heritable, suggesting potential for evolutionary responses to temperature changes (Logan and Cox 2020). Upper thermal limits may show comparatively slow divergence as organisms can avoid extreme heat exposure via behavioral thermoregulation, such as seeking thermal refugia, thereby leading to lower selective pressure on CTmax. However, if environmental temperatures exceed the physiological and behavioral buffering capacities of organisms, extinction rates can increase (Araújo et al. 2013; Gunderson and Stillman 2015; Bennett et al. 2021). At finer spatial scales, dissimilarity in habitat use and microclimate exposure can contribute to substantial differences in realized thermal niches, even among closely related taxa. In *Anolis* lizards, species occupying open habitats generally experience warmer and more thermally variable environments compared to species using closed canopy or shaded habitats. These differences in habitat use are linked to divergence in thermal physiology, including heat tolerance, and can influence climate vulnerability through differences in thermoregulatory opportunities (Gunderson and Leal 2012; Logan et al. 2013; Gunderson et al. 2018). Similar microhabitat-driven divergence occurs in Liolaemidae, where substrate selection strongly influences body temperatures and thermoregulatory behavior (Vivas et al. 2019). These differences consequently influence geographic distributions and climate vulnerability, with widespread species typically exhibiting greater behavioral flexibility and performance breadth, while habitat specialists often operate closer to their limits with narrower safety margins (Calosi et al. 2010; Slatyer et al. 2013; Diamond and  Chick 2018). In sympatric species that share the same local environment, the interaction between evolutionary history, microhabitat use, and behavioral thermoregulation may therefore produce differences in thermal resilience (Gunderson et al. 2018; Bodensteiner et al. 2021). Understanding how evolutionary constraints interact with ecological flexibility is therefore critical for predicting species-specific resilience to climate warming.

Despite being a global reptile diversity hotspot, the Indian subcontinent remains underrepresented in thermal ecology studies (but see Tatu et al. 2024; Apte et al. 2025). Here, we investigate thermal ecology and vulnerability in two co-occurring agamid lizards from semi-arid southern India with different microhabitat preferences and geographic distribution ranges; the arboreal generalist *Calotes versicolor*, with a broad distribution across South and Southeast Asia, and the saxicolous specialist *Psammophilus dorsalis*, endemic to peninsular India (Radder et al. 2005; Gowande et al. 2021). Although they coexist in the same habitats, their distinct structural niches suggest different thermal experiences. We asked whether these co-occurring species differ in the thermal environments they exploit, their thermoregulatory effectiveness, and their vulnerability to future warming. To understand this, we (1) measured environmental temperatures using physical copper models in open and shaded microhabitats and field body temperatures (Tb) of lizards; (2) quantified preferred temperatures (Tpref), critical thermal limits (CTmax and CTmin) and thermal performance curves of sprint speed and bite force; (3) calculated species-specific indices of thermal vulnerability, and (4) projected performance declines under three IPCC Shared Socioeconomic Pathways (SSP1-2.6, SSP2-4.5, and SSP5-8.5). We predicted that the rock-dwelling specialist, *P. dorsalis*, would exhibit higher optimal temperatures (Topt) for performance and steeper performance declines under warming, due to greater exposure to high temperatures in its microhabitat compared to the arboreal generalist, *C. versicolor* (Sunday et al. 2014). Consequently, we expect *P. dorsalis* to be more vulnerable to climate warming than *C. versicolor*. By comparing these co-occurring species, we disentangle the roles of environmental conditions, species physiology, and behavior in determining resilience to climate warming.  

## Methods

### Study area

The study was conducted from April to August in 2023 and 2024, coinciding with the peak activity period of these species, at three sites near Bangalore, Karnataka, India: Narasapura (Kolar- 13°07ʹ33.7″N 78°01ʹ07.3″E), Bannerghatta (Anekal- 12°48ʹ57.3″N 77°34ʹ34.4″E), and Avati (Devanahalli- 13°17ʹ51.3″N 77°42ʹ37.2″E). These areas consist of rocky outcrops interspersed with houses and agricultural fields. Across sites, air temperatures reach a maximum of approximately 39°C during the hottest month (April) and drop to a minimum of about 14°C during cooler periods. Annual rainfall is moderate, averaging roughly 950–960 mm a year [India Meteorological Department data (IMD) 2025]. Only male adult lizards were used in this study to minimize potential variation arising from sex-specific differences in body size and reproductive state (Gainsbury 2020).

### Physical model temperatures of microhabitats

To characterize microenvironmental temperatures experienced by the lizards, we deployed physical models in microhabitats utilized by these species (following general approaches in Bakken 1992; Dzialowski 2005; Bakken and Angilletta Jr 2014). Physical models were made using 31-gauge (0.295 mm) copper sheets as hollow cylinders with closed ends that mimic the dimensions of a typical adult lizard of both species (12 cm length and 9 cm maximum circumference). These physical models were equipped with a Thermochron iButton (Maxim Integrated, DS1921G) inside that recorded temperature at 2-min intervals. Laboratory calibration in a water bath showed a strong correlation between physical model temperatures and cloacal body temperatures of live lizards at thermal equilibrium (Supplementary Information 1a). Accordingly, field-recorded physical model temperatures for *C. versicolor* and *P. dorsalis* were corrected using species-specific calibration equations derived from these relationships (Supplementary Information 1a). However, the physical models exhibited greater thermal inertia than live lizards, so temperatures recorded by these models are a proxy for microenvironmental temperatures rather than true operative temperatures (Te) (see Supplementary Information 1b for details).

In the wild, generalist *C. versicolor* inhabits a wide range of habitat structures ranging from trees, scrub vegetation, concrete fence poles and stone walls. In contrast, *P. dorsalis* is restricted to predominantly rocky substrates like boulders, stone walls and poles. To capture the variation in temperature across the range of microhabitats used by both species, we deployed the physical models in two broad microhabitat categories: open and shaded. Open microhabitats comprised sun- exposed microhabitats, including rocks, ground, fence poles, and stone walls, whereas shaded microhabitats included shaded rocks, crevices, tree trunks, and shaded poles and walls. Sampling was conducted across three regions (Kolar, Bannerghatta, and Avati) during the peak summer months (April–June 2024). At each site and each month, three models were placed in each microhabitat type, resulting in a total of 27 models per site per month. Models were spaced approximately 100 m apart and deployed continuously for three consecutive days.

### Field body temperature

In parallel with physical model deployment, adult male lizards (*C. versicolor; n* = 53, *P. dorsalis; n* = 152) were opportunistically captured by lassoing from dawn to dusk to obtain field-active body temperatures. Body temperatures (Tb) were measured immediately upon capture using a calibrated Type-K thermocouple (Amprobe TMD 50) inserted approximately 10 mm into the cloaca. Lizards were handled gently using a cloth bag to minimize stress, and body temperatures (Tb) were measured within 1 min of capture. All procedures were non-lethal, and lizards were released unharmed at the location of capture immediately following data collection. Snout to vent length (SVL) using a ruler and body mass using a portable weighing scale were recorded for each lizard. Body mass and SVL were used to calculate Scaled Mass Index as a measure of body condition of lizards, as per (Peig and Green 2009).

### Capture and housing

Adult male lizards were captured by lassoing from the wild and transported to the laboratory within 12 hours of capture. During transport, lizards were held in individual cloth bags and placed in a cooler with icepacks to minimize stress. Each lizard was housed individually in a glass terrarium measuring 60 × 30 × 25 cm (L × W × H) in the lizard housing facility maintained at the Centre for Ecological Sciences, Indian Institute of Science, Bangalore. The housing room was maintained on a 12:12 h light:dark photoperiod (lights on 0700–1900 h) at an ambient temperature of 25–27°C. Illumination was provided using standard white room lighting in visible spectrum. Terraria were lined with disposable paper towels as substrate and contained a rock to perch and a PVC pipe as a refuge. Each terrarium was equipped with an incandescent basking lamp (60 W) positioned at one end to create a thermal gradient within the enclosure, allowing lizards to behaviorally thermoregulate. Terraria were cleaned and paper towels were replaced every 3–4 days, or more frequently if soiled. Lizards had *ad libitum* access to water and were fed mealworms daily. Visual contact between individuals was minimized by using opaque barriers between terraria. All lizards were allowed to acclimate for 48 h prior to the start of experiments. All individuals were released at their exact site of capture within 7 days of capture.

### Preferred temperature range

Preferred temperature (Tpref) of *C. versicolor* (*n* = 24) and *P. dorsalis* (*n* = 38) were measured using a wooden linear thermal gradient comprised of 4 separate tracks (200 cm length × 12 cm width × 40 cm height), lined with a 320-grit sandpaper substrate to provide traction. The thermal gradient ranged from approximately 8 to 52°C, achieved with ice packs at one end and ceramic heat lamps at the other. Body temperature (Tb) of lizards was measured using a Type-K thermocouple (Amprobe model TMD-50), which was inserted ∼10 mm into the cloaca and secured to the base of the tail using a surgical tape. Lizards were provided with food the evening before the trials but were not fed on the day of trials. Lizards were placed individually on the track and were allowed to freely move. After a 1-h acclimation period, Tb was recorded every 10 min for the following 2 h. The Tpref for each individual was calculated as the median of all recorded Tb values during the observation period. These same individuals were subsequently used to determine critical thermal limits, following a minimum of 24 h of rest after the Tpref trial.

### Critical thermal limits

Critical thermal limits (CTmax and CTmin) were measured as the body temperatures at which individuals lost the righting response (i.e., failed to turn over when placed on their backs). The order of CTmax and CTmin was randomized across individuals. CTmax data were collected for 24 *C. versicolor* and 38 *P. dorsalis* and CTmin data for 22 *C. versicolor* and 37 *P. dorsalis*. Lizards that showed slow recovery or died (*n* = 3) after the first trial were not included in the following trials, resulting in differences in sample sizes between CTmax and CTmin measurements. Cloacal temperature (Tb) was recorded using a Type-K thermocouple (Amprobe TMD-50), inserted 10 mm into the cloaca and secured to the base of the tail with surgical tape. All trials began with lizards at an initial body temperature of ∼27°C. To measure CTmax, each lizard was placed inside a stainless-steel container immersed in a water bath to ensure uniform heating. The water bath temperature was increased to raise Tb at ∼1°C per minute. Righting response tests were initiated once the lizard looked visibly sluggish and repeated every minute until the lizard failed to right itself (Labra et al. 2001). CTmin was determined using the same procedure but by cooling the water bath with ice to decrease Tb at ∼1°C per minute. Critical thermal limits of each lizard were tested on separate days, with a minimum of 24 h between trials, to prevent thermal stress or carryover effects. Thermal tolerance range (TTR) was calculated as the difference between upper and lower critical thermal limits of the lizards. (TTR = CTmax—CTmin).

### Bite force and sprint speed

The performance trials were measured using a separate set of individuals (*C. versicolor; n* = 42, *P. dorsalis*; n = 42) from those used in the Tpref and critical thermal limits to minimize repeated handling stress and fatigue effects on lizards (following Pontes-da-Silva et al. 2018; Apte et al. 2025). To examine how physiological performance varies with temperature, bite force and sprint speed were measured across a range of ecologically relevant body temperatures. Following acclimation of 48 h in lizard room at 25–26°C, lizards were placed in a programmable incubator (Vevor Reptipro 6000) and allowed to attain one of the following body temperatures—15, 20, 25, 30, 35, and 40°C. Body temperature (Tb) was monitored using cloacal thermocouple and the desired temperature was achieved within 15–20 min once placed in the incubator. All performance measurements were conducted in an experiment room maintained at 26°C, within 1–3 min after the individuals achieved the target temperature. Re-measuring the Tb after the trial confirmed that Tb remained close to the experiment temperature. Each individual lizard was tested at only one temperature. Each temperature treatment included 7 individuals (total *n* = 42 per species and 6 temperatures).

Bite force was measured using a custom-built force transducer (Amdekar et al. 2022). At each temperature treatment, once the lizard reached the desired Tb, it was induced to bite on the sensor plates of the force meter. Three successive trials were conducted for an individual, and the highest bite force value from the three trials was retained for analysis. The lizard was placed back in the incubator for 10 min, before testing sprint speed.

Sprint speed was measured in a 320-grit sandpaper lined 3-m-long racetrack, divided visually into 0.25-m segments. Lizards were encouraged to run the length of the track by tapping their tail using a brush, and each trial was video recorded using a GoPro camera (120 fps) wall-mounted on the top of the racetrack. Sprints were repeated twice, separated by a 10 min rest period, during which lizards were kept in the incubator set at the experiment temperature. The fastest speed over a single interval was calculated by frame-by-frame analysis using Avidemux 2.7.8 software (https://avidemux.sourceforge.net/), and the maximum value across trials was used for thermal performance curve construction.

### Thermal vulnerability indices

Using thermal traits measured in the laboratory and the microhabitat temperature data collected in the wild using physical models, various thermal indices were calculated to quantify species-specific vulnerability to climate warming. These indices were computed separately for each species across open and shaded microhabitats to assess interspecific differences in thermal buffering capacity and potential climate sensitivity. We calculated thermoregulatory accuracy (db), as the absolute deviation of field-active body temperature from the laboratory-derived preferred temperature (Tb − Tpref), following Hertz et al. (1993), with smaller values indicating better thermoregulatory accuracy. If Tb was below the Tpref range, we subtracted Tb from the lower bound of Tpref range. If Tb was above the Tpref range, we subtracted upper bound of Tpref from Tb. Thermal quality of habitat (de) was calculated as the absolute deviation of microhabitat temperature measured using physical models from the preferred temperature range (Microhabitat temperature − Tpref). When microhabitat temperatures fell within the preferred temperature range, deviation was assigned a value of zero. If the microhabitat temperature was below the Tpref range, we subtracted it from the lower bound of the Tpref range and if it was above the Tpref range, we subtracted the upper bound of Tpref from the microhabitat temperature. The smaller values indicate higher habitat thermal quality, following Hertz et al. (1993). We calculated the effectiveness of thermoregulation (E) as the index of behavioral thermoregulation relative to the thermal quality of the habitat $(1 - (\overline {{d}_b} /\overline {{d}_e} ))$. Values of E approaching 1 indicate highly effective thermoregulation, where individuals maintain body temperatures close to their preferred range despite low thermal habitat quality. Values near 0 indicate thermoconformity, where body temperatures largely reflect environmental conditions. Negative values of E indicate ineffective thermoregulation, with individuals maintaining body temperatures farther from preferred temperatures than expected based on habitat thermal quality alone (Hertz et al. 1993; Blouin-Demers and Weatherhead 2001). Warming tolerance (WT) was calculated as the difference between the critical thermal maximum and the mean field-active body temperature (CTmax − mean Tb) (Brusch et al. 2016). WT indicates the temperature change an organism can tolerate before the environment is no longer suitable (Deutsch et al. 2008); lower WT values indicate reduced capacity to tolerate further warming. The thermal safety margin (TSM) was calculated as the absolute buffer between the critical thermal maximum and the maximum temperature in open microhabitats measured using physical models (CTmax − max open microhabitat temperature) (following Sunday et al. 2014). TSM represents the thermal buffer between current microhabitat temperatures and upper physiological limits, with smaller values indicating greater vulnerability to future warming.

### Statistical analyses

All statistical analyses were conducted in R (R Core Team 2024). Unless otherwise stated, we fitted linear mixed-effects models with location (Avati, Bannerghatta, and Kolar) included as a random intercept to control for potential spatial non-independence. Model assumptions, including normality of residuals and homoscedasticity, were verified visually using diagnostic plots for all analyses.

To characterize the thermal environment of microhabitats for the two species, we modeled microhabitat temperature measured with physical models using the lme4 package (Bates et al. 2015). The microhabitat temperature was log-transformed to meet normality assumptions and modeled as a function of species, microhabitat (open vs. shade), and their interaction. Date was included as an additional random intercept to account for temporal variation. Pairwise comparisons were conducted using estimated marginal means in the emmeans package, back-transformed to the original scale for interpretation. To determine the drivers of field body temperature (Tb), we modeled Tb as a function of species, microhabitat temperatures in open and shade at the same time as that of Tb, their interactions with species and BCI as fixed effects.

Tpref, CTmax and CTmin were compared between the two species using generalized linear models (GLMs) with a Gaussian error distribution and identity link function. Species was included as a fixed effect in each model. Separate models were fitted for Tpref, CTmax, and CTmin. Model assumptions were evaluated by visual inspection of residual plots and tests of residual normality. Statistical significance was assessed using analysis of deviance with F-tests.

To characterize the thermal sensitivity of bite force and sprint speed, we fitted generalized additive mixed models (GAMMs) using the mgcv package (Wood and Wood 2015). We modeled performance as a function of Tb (using a smooth spline with *k* = 4 basis functions) and BCI, with location included as a random intercept. Separate models were fitted for each species and performance traits. From these fitted models, we estimated thermal optimum (Topt), maximum performance (Pmax), and 80% performance breadth (B80) (Hertz et al. 1993). Optimum temperature (Topt) was the body temperature at which the lizard’s speed was maximum (Pmax). Performance breath (B80) was defined as the range of Tb at which the performance is greater than or equal to 80% of Pmax. The performance curves were constrained to zero performance at the species-specific critical thermal limits (CTmin and CTmax).

To assess species differences in thermoregulatory accuracy (db), we fitted a linear model with species as the fixed effect. For habitat thermal quality (de) and effectiveness of thermoregulation (E), we constructed separate linear models including species, microhabitat (open vs. shade), and their interaction as fixed effects. Significant interactions were resolved using post-hoc pairwise contrasts to identify specific species-by-microhabitat differences.

### Climate projection

To predict potential effects of climate warming on sprint speed and bite force, we followed the general framework of Neel et al. (2021). Air temperature projections were generated from 2024 to 2100 under three IPCC AR6 Shared Socioeconomic Pathways (SSPs; IPCC 2021): SSP1-2.6 (+0.6°C total warming; +0.008°C year⁻¹), SSP2-4.5 (+1.5°C; +0.020°C year⁻¹), and SSP5-8.5 (+3.2°C; +0.042°C year⁻¹). We assumed that projected increases in mean air temperature under each SSP translated proportionally into increases in environmental temperatures measured using physical models in open habitats, representing warming experienced by lizards in exposed environments. Species-specific relationships between Tb and environmental temperatures were obtained from the model incorporating species × microhabitat interactions. These relationships were then used to estimate how projected warming in open habitats would translate into future changes in body temperature for each species, given their current thermoregulation. The resulting projected body temperatures were subsequently applied to species- and trait-specific thermal performance curves fitted using GAMMs to estimate future sprint speed and bite force performance. Current performance at each species’ thermal optimum (Topt) was standardized to 100%, and projections assumed no evolutionary adaptation, phenotypic plasticity, or shifts in thermal limits (Tatu et al. 2024).

## Results

### Physical model temperatures of microhabitats

**Fig. 1:**
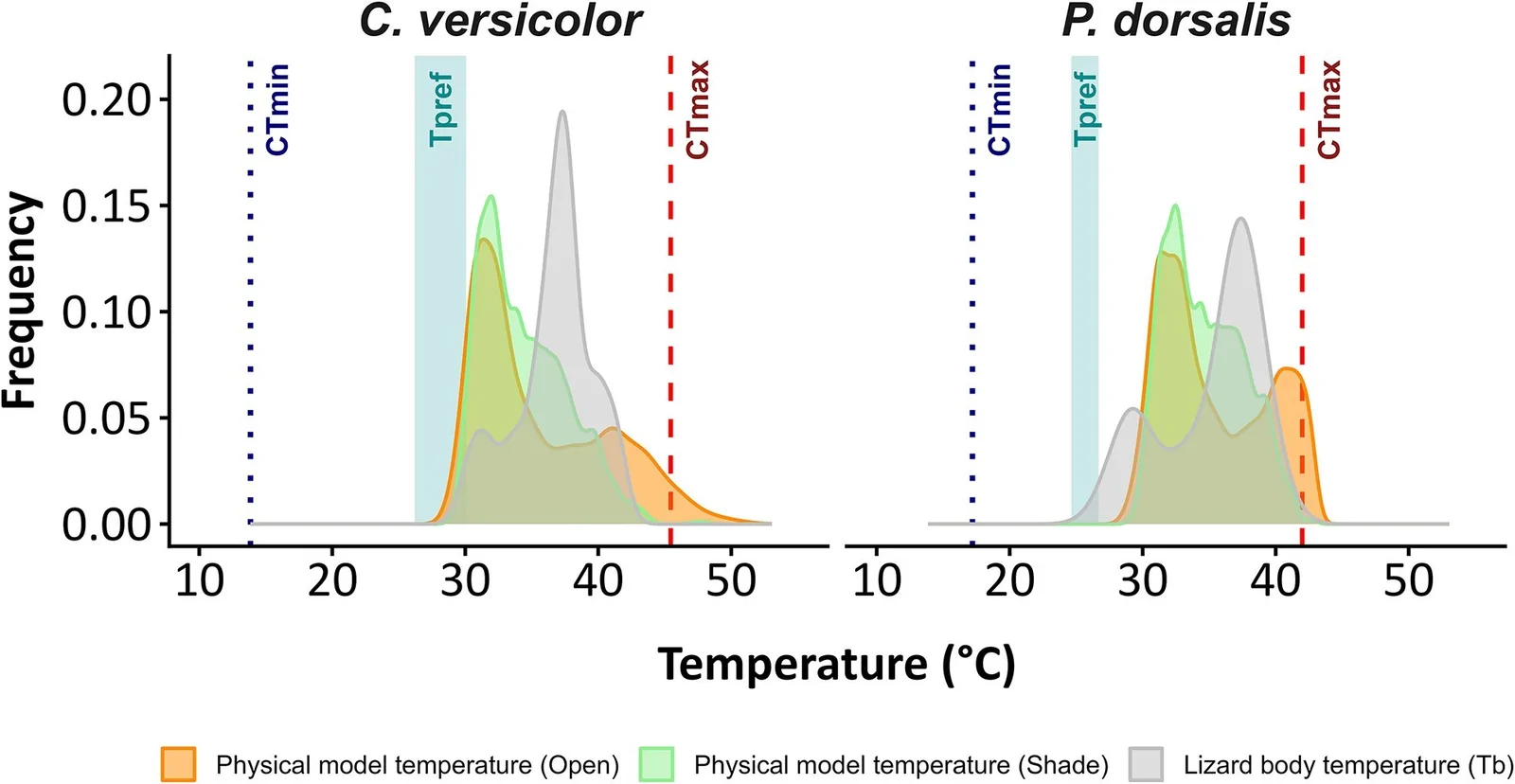
Frequency distributions of microhabitat temperatures measured using physical models and field body temperatures (Tb) relative to physiological limits for *C. versicolor* (A) and *P. dorsalis* (B). Density plots illustrate the temperature availability in open and shaded microhabitats compared to the realized field body temperatures of each species. The figure also shows the species-specific preferred temperature ranges (Tpref), and critical thermal minima (CTmin) and maxima (CTmax) for each species.

Physical model temperatures in the wild were explained by a significant interaction between species and microhabitat (β = 0.0167, SE = 0.0011, *t* = 15.54, *P* < 0.001, Fig. S2a). Within open microhabitats, physical model temperatures were significantly higher for *C. versicolor* than *P. dorsalis* (β = 0.0115, SE = 0.0008, *z* = 14.24, *P* < 0.001, Table 1). Microhabitat temperature in the shade was significantly lower than temperatures in open microhabitats for both species (Fig. 1, Table 1). For *C. versicolor*, the difference in temperature for shade and open microhabitats was 1.5°C (β = 0.0418, SE = 0.0008, *z* = 54.87, *P* < 0.001) and for *P. dorsalis*, it was 0.9°C (β = 0.0251, SE = 0.0008, *z* = 32.89, *P* < 0.001, Table 1). Within shade microhabitats, *P. dorsalis* experienced slightly but significantly higher temperatures than *C. versicolor* by 0.2°C (β = −0.0053, SE = 0.0007, *z* = −7.38, *P* < 0.001). Random effects (location and date) collectively explained 17% of the total variance (Date: σ² = 0.0022; Location: σ² = 0.00045).

**Table 1:** Summary of thermal indices, performance metrics, and vulnerability projections for *C. versicolor* and *P. dorsalis*.

Indices	Units		*C. versicolor*	*P. dorsalis*
Microhabitat temperature using physical models	(°C)	Open	35.79 (95% CI: 34.65–36.97)	35.38 (95% CI: 34.25–36.54)
		Shade	34.32 (95% CI: 33.23–35.45)	34.50 (95% CI: 33.40–35.64)
Preferred temperature range	Tpref (°C) (Mean ± SD)		28.23 ± 3.57 (Range: 26.21–30.06) (*n* = 24)	25.35 ± 2.17 (Range: 24.63–26.71) (*n* = 38)
Critical thermal maximum	CTmax (°C) (Mean ± SD)		45.45 ± 1.69 (*n* = 24)	42.00 ± 2.04 (*n* = 38)
Critical thermal minimum	CTmin (°C) (Mean ± SD)		13.85 ± 1.30 (*n* = 22)	17.20 ± 2.36 (*n* = 37)
Thermal tolerance range	TTR (°C) (Mean ± SD)		31.5 ± 2.17	25.1 ± 3.91
Performance	Bite force	Pmax (KgF)	3.72 (*n* = 42)	2.65 (*n* = 42)
		Topt (°C)	26.51	27.27
		B80	20.14–34.01 (13.87)	20.11–34.81 (14.70)
	Sprint speed	Pmax (m/s)	2.32 (*n* = 42)	2.54 (*n* = 42)
		Topt (°C)	34.01	33.02
		B80	27.64–39.63 (11.99)	26.92–38.39 (11.47)
Accuracy of thermoregulation	db = Tb—Tpref (Mean ± SD)		6.53 ± 2.52	8.96 ± 2.94
Thermal quality of habitat	de = Microhabitat temperature − Tpref (Mean ± SD)	Open	10.89 ± 3.66	12.28 ± 2.83
		Shade	6.21 ± 2.81	9.59 ± 2.69
Effectiveness of thermoregulation	E = 1 − (db/de)	Open	0.40	0.28
		Shade	−0.15	0.06
Warming Tolerance	WT = CTmax − Mean Tb		8.68	6.21
Thermal Safety Margin	TSM = CTmax − Maximum open microhabitat temperature		−4.87	−0.82
Projection	Bite force	SSP1-2.6	0.01%	0.06%
		SSP2-4.5	0.06%	0.37%
		SSP5- 8.5	0.31%	1.73%
	Sprint speed	SSP1-2.6	0%	0.1%
		SSP2-4.5	0.04%	0.65%
		SSP5- 8.5	0.36%	3.09%

*Note:* Shown are estimates for temperatures in open and shade microhabitats measured using physical models, preferred temperature range (Tpref) and critical thermal limits (CTmin and CTmax), and thermal sensitivity [Maximum performance (Pmax), Optimal temperature (Topt) and 80% performance breadth (B80)] of bite force and sprint speed. Also included are indices of thermoregulatory efficiency (accuracy, habitat quality, and effectiveness) and thermal vulnerability (WT and TSMs). Future risk is represented by percentage declines in performance projected under Shared Socioeconomic Pathways (SSP1-2.6, SSP2-4.5, and SSP5-8.5). Sample sizes are indicated as *n* = .

### Body temperature

The relationship between Tb and microhabitat temperatures differed between species, as indicated by significant species × open microhabitat temperature (β = 0.41, SE = 0.14, *t* = 2.97, *P* = 0.003) and species × shade microhabitat interactions (β = −0.36, SE = 0.18, *t* = −2.06, *P* = 0.041). BCI had no significant effect on Tb (β = 0.007 ± 0.018 SE, *P* = 0.68), while variance explained by location was negligible. Post hoc slope estimates showed that *C. versicolor* responded to both open and shaded microhabitat temperatures, with Tb increasing by 0.30°C (95% CI: 0.11–0.50) per 1°C increase in microhabitat temperature in open and by 0.44°C (95% CI: 0.17–0.71) in shade. In contrast, *P. dorsalis* exhibited a stronger relationship with microhabitat temperature in open (slope = 0.72, 95% CI: 0.52–0.91), whereas the relationship was weak and non-significant in shade (slope = 0.08, 95% CI: −0.15–0.30).

### Tpref, CTmax, and CTmin

*Psammophilus dorsalis* exhibited significantly lower preferred body temperatures (Tpref) than *C. versicolor* (GLM: *F*_1,60_ = 16.07, *P* < 0.001). The estimated Tpref of *P. dorsalis* was approximately 2.91°C lower than that of *C. versicolor* (Table 1). Critical thermal maximum (CTmax) also differed significantly between species (*F*_1,60_ = 51.92, *P* < 0.001), with *P. dorsalis* exhibiting a lower CTmax than *C. versicolor* by approximately 3.60°C (Table 1). In contrast, critical thermal minimum (CTmin) was significantly higher in *P. dorsalis* than in *C. versicolor* (*F*_1,57_ = 28.02, *P* < 0.001). *Psammophilus dorsalis* had CTmin values approximately 2.90°C higher than those of *C. versicolor* (Table 1). Overall, *C. versicolor* exhibited a broader TTR (mean ± SD = 31.5 ± 2.17°C) than *P. dorsalis* (25.1 ± 3.91°C), reflecting its higher upper thermal limits and lower thermal limits (Table 1).

### Thermal performance curves

**Fig. 2:**
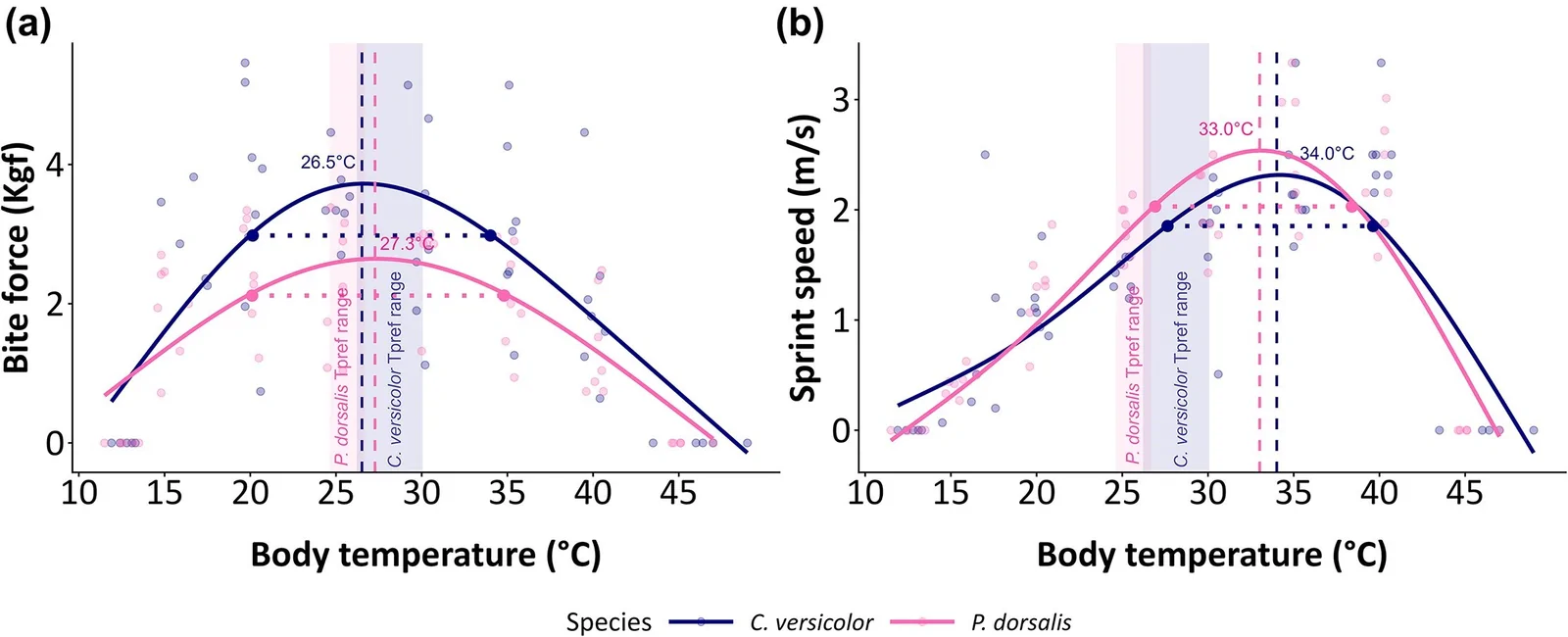
Thermal performance curves for bite force and sprint speed for *C. versicolor* and *P. dorsalis*. Solid lines represent the fitted performance curves (GAMM) relative to body temperature (Tb), while filled circles denote individual trial data. Vertical dashed lines indicate the thermal optimum (Topt), the temperature at which bite force or sprint speed is maximized. Horizontal dotted segments are the 80% performance breadth (B80), representing the thermal range within which performance exceeds 80% of the maximum. Shaded vertical bands highlight the preferred body temperature (Tpref) range for each species.

Thermal sensitivity of performance varied by trait and species (Fig. 2). For bite force, the smooth terms for body temperature were significant for both *C. versicolor* (*F* = 13.51, *P* < 0.001) and *P. dorsalis* (*F* = 16.58, *P* < 0.001), although the thermal optima (26.5°C for *C. versicolor* and 27.3°C for *P. dorsalis*) were relatively low and aligned closely with preferred temperatures. For sprint speed, the smooth terms for body temperature were significant for both *C. versicolor* (*F* = 32.28, *P* < 0.001) and *P. dorsalis* (*F* = 88.64, *P* < 0.001). Thermal optima (Topt) for sprint speed (34.01°C for *C. versicolor* and 33.02°C for *P. dorsalis*) were notably higher than preferred temperatures (26.21–30.06°C for *C. versicolor* and 24.63–26.68°C for *P. dorsalis*) for both species. The 80% performance breadth (B80) for bite force was similar between species (Table 1, Fig. 2). For sprint speed, B80 was also comparable between species but narrower than that of bite force (Table 1, Fig. 2). Random effects (location) explained substantial variance for *P. dorsalis* performance (bite force: σ² = 0.13; sprint speed: σ² = 0.08), but negligible variance for *C. versicolor* (bite force: σ² = 0.03; sprint speed: σ² < 0.001). BCI had no effect on both performance metrics for both species.

### Thermal vulnerability indices

Body temperature deviation from the preferred temperature range (db) differed significantly between species (*F*₁,₂₀₃ = 28.45, *P* < 0.001), with *P. dorsalis* exhibiting a higher mean deviation compared to *C. versicolor* (Table 1). The thermal quality of the habitat (de) was explained by a significant interaction between species and microhabitat type (*F*₁,₄₀₆ = 9.18, *P* = 0.0026). Both species experienced significantly lower de values in shaded microhabitats compared to open microhabitats (Table 1, *C. versicolor; t*₄₀₆ = 8.23, *P* < 0.001, *P. dorsalis; t*₄₀₆ = 8.10, *P* < 0.001). Between species, *P. dorsalis* showed significantly higher de values than *C. versicolor* in both open (*t*₄₀₆ = −2.99, *P* = 0.016) and shaded microhabitats (*t*₄₀₆ = −7.28, *P* < 0.001, Table 1). Similarly, the effectiveness of thermoregulation (E) was driven by a significant interaction between species and microhabitat (*F*₁,₄₀₆ = 22.27, *P* < 0.001). While thermoregulatory effectiveness varied across microhabitats, *C. versicolor* and *P. dorsalis* showed higher effectiveness in open microhabitats compared to the shade (*C. versicolor; t*₄₀₆ = 9.27, *P* < 0.001, *P. dorsalis; t*₄₀₆ = 6.54, *P* < 0.001, Table 1). WT was higher in *C. versicolor* compared to *P. dorsalis* and TSM was negative for both species in open microhabitats (Table 1).

### Climate projection

Climate projections incorporating species-specific Tb responses to environmental temperatures measured using physical models indicated limited impacts of warming on sprint speed and bite force across all scenarios. Under low-emission (SSP1-2.6) and intermediate-emission (SSP2-4.5) scenarios, projected declines remained below 1% for both traits in both species (Table 1). Under the high-emission SSP5-8.5 scenario, *C. versicolor* showed an estimated Tb increase of 0.97°C by 2100, resulting in only minor reductions in performance (bite force: 0.31%; sprint speed: 0.36%). In contrast, *P. dorsalis* exhibited a projected Tb increase of 2.28°C, leading to declines in bite force (1.73%) and sprint speed (3.09%). Sprint speed was consistently more sensitive to warming than bite force in both species, reflecting its narrower thermal breadth and higher thermal optimum (Table 1).

## Discussion

Thermal traits of co-occurring ectotherms are shaped by their evolutionary history, physiology, behavior, and microhabitat use. In this study, two co-occurring agamid lizards, *P. dorsalis* and *C. versicolor*, occupying the same semi-arid landscape, showed both similarities and differences in their thermal traits. Both species are exposed to similar temperatures in open microhabitats during peak summer and their thermal performance breadths (B₈₀) for sprint speed and bite force were broadly similar. These commonalities likely reflect similarities in body size, phylogenetic history within Agamidae, and adaptation to warm semi-arid environments (Angilletta Jr. 2009; Rubalcaba and Olalla-Tárraga 2020). However, clear interspecific differences emerged in thermal physiology and vulnerability indices. *Psammophilus dorsalis* exhibited significantly lower preferred body temperatures (Tpref) than *C. versicolor*, lower critical thermal maximum (CTmax), and higher critical thermal minimum (CTmin), resulting in a narrower overall TTR. These physiological differences align with the more restricted saxicolous niche and narrower geographic distribution of *P. dorsalis*, while the broader tolerance of *C. versicolor* is consistent with its wide-ranging arboreal generalist ecology. Together, these patterns indicate that co-occurring species can occupy distinct thermal niches despite experiencing similar average climatic conditions in shared landscapes.

Microhabitat temperatures, measured through physical models, differed between open and shaded microhabitats for both species, but the contrast was modest during daytime peak summer conditions. This likely reflects the reduced thermal buffering capacity of shaded microhabitats during hot summers in this semi-arid region. Similar reductions in the thermoregulatory effectiveness of shaded microhabitats under extreme heat have been reported in Mediterranean lizards and in other taxa (Lima et al. 2016; Díaz et al. 2022). Despite the relatively small thermal differences between microhabitats, the two species differed in how these temperatures translated into body temperatures (Tb). *Psammophilus dorsalis* showed a stronger relationship between Tb and temperatures in open microhabitats, whereas *C. versicolor* responded to both open and shaded microhabitats. This pattern likely reflects differences in habitat use, with *P. dorsalis* commonly occupying exposed rocky substrates and *C. versicolor* using more structurally complex arboreal habitats that may provide greater thermal buffering. These differences subsequently influenced climate projections, with warming translating into larger future Tb increases in *P. dorsalis*. Similar effects of habitat thermal structure and thermoregulatory opportunities on thermal exposure and climate vulnerability have been reported in Anolis lizards, where access to heterogeneous microhabitats seem to buffer future warming impacts and maintain performance levels (Neel et al. 2021).

Thermal performance curves differed between sprint speed and bite force but were similar across both species, with each trait showing comparable thermal optima. For both species, sprint speed was optimal at a higher body temperature and had a narrower performance breadth than bite force, indicating greater thermal sensitivity of locomotion (Herrel et al. 2007; Angilletta 2009). Optimal temperature for bite force was closer to the preferred temperature for both species, but the optimal temperature for sprint speed was much higher than the Tpref. This mismatch is consistent with theoretical expectations for thermoregulation under asymmetric thermal performance curves. As performance curves typically decline more steeply above than below Topt, the optimal strategy for maximizing overall fitness is often to maintain body temperatures below the mean Topt of multiple performance traits, particularly when thermoregulation is imperfect (Martin and Huey 2008; Kubisch et al. 2011). Our results support this pattern, suggesting that thermoregulation in these agamid lizards balances multiple fitness-related functions rather than maximizing any single performance trait, such as locomotor speed (similar to Tatu et al. 2024).

Indices of thermal vulnerability revealed interspecific differences in how the two species experience and respond to their thermal environments. *Psammophilus dorsalis* showed larger deviations of body temperature from preferred temperatures and lower habitat quality than *C. versicolor*, indicating reduced thermoregulatory accuracy (Blouin-Demers and Nadeau 2005). Although both species exhibited higher thermoregulatory effectiveness in open microhabitats than shade, *P. dorsalis* consistently operated closer to its upper thermal limits and exhibited lower WT than *C. versicolor*, indicating greater vulnerability to future warming. Similar patterns have been reported in tropical lizards, where species with restricted microhabitat use or limited behavioral buffering show reduced thermoregulatory effectiveness and elevated vulnerability indices (Valenzuela-Ceballos et al. 2015; Medina et al. 2016). Zero or negative TSMs are increasingly reported in tropical ectotherms and are considered predictors of climate-related risk (Deutsch et al. 2008; Sunday et al. 2014).

Our projections indicate minimal potential effects of climate warming on sprint speed and bite force performance under any of the emission scenarios (SSP1-2.6, SSP2-4.5, SSP5-8.5). Sprint speed was more thermally sensitive than bite force in both species, with higher thermal optima and narrower performance breadths, but overall performance impacts from uniform temperature shifts appear limited. These findings parallel results from Bahamian forest-edge Anolis lizards, where projected performance levels were largely retained by the end of century under explicit consideration of thermoregulatory opportunities, in contrast to stronger declines projected for Panamanian forest species (Neel et al. 2021). Nevertheless, the lower WT and TSM of *P. dorsalis*, combined with its saxicolous specialization and reduced thermoregulatory accuracy, suggest potential vulnerability through ecological pathways not captured by average performance projections. Narrow TSMs can restrict activity time through increased refuge use, elevate energetic costs, and increase vulnerability to extreme thermal events even when mean performance remains relatively stable (Sunday et al. 2014).

Our results align with broader patterns in tropical lizard thermal ecology, where closely related species occupying different microhabitats diverge in vulnerability despite regional climate similarity (Gunderson and Leal 2012; Guerra-Correa et al. 2020). Importantly, our projections assume uniform shifts in temperatures and do not fully incorporate behavioral plasticity, potential acclimation, or evolutionary adaptation. Although *P. dorsalis* already encounters high environmental temperatures, individuals may behaviorally exploit cooler refuges (e.g., crevices or shaded rocks) to reduce exposure, and we lack direct data on the plasticity or heritability of CTmax in these populations. Projected changes in thermal buffering provided by shaded microhabitats were also not incorporated. Future work should quantify operative temperature distributions at finer scales, and genetic variation in thermal traits to better predict long-term outcomes.

Overall, our study illustrates that co-occurring tropical lizards can occupy distinct thermal niches shaped more by microhabitat specialization than by broad environmental exposure alone. While direct impacts on key performance traits such as sprint speed and bite force may be modest under many climate scenarios, the combination of narrow WTs, safety margins, and habitat specialization, particularly for *P. dorsalis*, suggests potential vulnerability via other ecological and physiological pathways. These findings contribute to the growing body of multi-species thermal ecology research in understudied tropical regions and emphasize the need for nuanced, trait- and habitat-specific assessments of climate risk in ectotherms.

## Ethics statement

The study was carried out under animal ethics clearance (*CAF/ETHICS/846/2021*) granted by the Institutional Animal Ethics Committee of the Indian Institute of Science.

## Supplementary Material

obag041_Supplemental_Files

## Data Availability

All the data used for the analyses are included in the electronic supplementary material.

## References

[bib1a] Amdekar MS, Thaker M. 2022. Colours of stress in male Indian rock agamas predict testosterone levels but not performance. Horm Behav 144:105214. 10.1016/j.yhbeh.2022.105214 35696781

[bib1] Angilletta MJ Jr . 2009. Thermal adaptation: A theoretical and empirical synthesis. Oxford, UK: Oxford University Press. 10.1093/acprof:oso/9780198570875.001.1

[bib2] Apte V, Tatu A, Thaker M. 2025. Microhabitat level thermal physiology and thermoregulation of a diurnal gecko in an urban landscape. Front Amphib Reptile Sci 2:1522805. 10.3389/famrs.2024.1522805

[bib3] Araújo MB, Ferri-Yáñez F, Bozinovic F, Marquet PA, Valladares F, Chown SL. 2013. Heat freezes niche evolution. Ecol Lett 16:1206–19. https://onlinelibrary.wiley.com/doi/10.1111/ele.12155 23869696 10.1111/ele.12155

[bib4] Bakken GS . 1992. Measurement and application of operative and standard operative temperatures in ecology. Am Zool 32:194–216. 10.1093/icb/32.2.194

[bib5] Bakken GS, Angilletta MJ Jr. 2014. How to avoid errors when quantifying thermal environments. Funct Ecol 28:96–107. 10.1111/1365-2435.12149

[bib6] Bates D, Maechler M, Bolker B, Walker S, Christensen RHB, Singmann H, Bolker MB. 2015. Package ‘lme4’. Convergence 12:2. https://github.com/lme4/lme4/

[bib7] Bennett JM, Sunday J, Calosi P, Villalobos F, Martínez B, Molina-Venegas R, Olalla-Tárraga MÁ, Algar AC, Singer A, Rahbek C et al. 2021. The evolution of critical thermal limits of life on Earth. Nat Commun 12:1198. https://www.nature.com/articles/s41467-021-21263-8 33608528 10.1038/s41467-021-21263-8 PMC7895938

[bib8] Blouin-Demers G, Nadeau P. 2005. The cost–benefit model of thermoregulation does not predict lizard thermoregulatory behavior. Ecology 86:560–6. 10.1890/04-1403

[bib9] Blouin-Demers G, Weatherhead PJ. 2001. Thermal ecology of black rat snakes (*Elaphe obsoleta*) in a thermally challenging environment. Ecology 82:3025–43. 10.1890/0012-9658(2001)082[3025:TEOBRS]2.0.CO;2

[bib10] Bodensteiner BL, Agudelo-Cantero GA, Arietta AZA, Gunderson AR, Muñoz MM, Refsnider JM, Gangloff EJ. 2021. Thermal adaptation revisited: how conserved are thermal traits of reptiles and amphibians? J Exp Zool Part A: Ecol Integr Physiol 335:173–94. 10.1002/jez.2414 32970931

[bib11] Brusch IV, G. A, Taylor EN, Whitfield SM. 2016. Turn up the heat: thermal tolerances of lizards at La Selva, Costa Rica. Oecologia 180:325–34. 10.1007/s00442-015-3467-3 26466592

[bib13] Calosi P, Bilton DT, Spicer JI, Votier SC, Atfield A. 2010. What determines a species’ geographical range? Thermal biology and latitudinal range size relationships in European diving beetles (Coleoptera: dytiscidae). J Anim Ecol 79:194–204. 10.1111/j.1365-2656.2009.01611.x 19761459

[bib14] Deutsch CA, Tewksbury JJ, Huey RB, Sheldon KS, Ghalambor CK, Haak DC, Martin PR. 2008. Impacts of climate warming on terrestrial ectotherms across latitude. Proc Natl Acad Sci 105:6668–72. 10.1073/pnas.0709472105 18458348 PMC2373333

[bib15] Diamond SE, Chick LD. 2018. Thermal specialist ant species have restricted, equatorial geographic ranges: implications for climate change vulnerability and risk of extinction. Ecography 41:1507–9. 10.1111/ecog.03264

[bib16] Díaz JA, Izquierdo-Santiago R, Llanos-Garrido A. 2022. Lizard thermoregulation revisited after two decades of global warming. Funct Ecol 36:3022–35. https://besjournals.onlinelibrary.wiley.com/doi/full/10.1111/1365-2435.14192

[bib17] Dzialowski EM . 2005. Use of operative temperature and standard operative temperature models in thermal biology. J Therm Biol 30:317–34. 10.1016/j.jtherbio.2005.01.005

[bib18] Gainsbury AM . 2020. Influence of size, sex, and reproductive status on the thermal biology of endemic Florida scrub lizards. Ecol Evol 10:13080–6. 10.1002/ece3.6897 33304518 PMC7713916

[bib19] Gowande G, Pal S, Jablonski D, Masroor R, Phansalkar PU, Dsouza P, Jayarajan A, Shanker K. 2021. Molecular phylogenetics and taxonomic reassessment of the widespread agamid lizard *Calotes versicolor* (Daudin, 1802) (Squamata, Agamidae) across South Asia. Vertebr Zool 71:669–96. 10.3897/vz.71.e62787

[bib20] Guerra-Correa ES, Merino-Viteri A, Andrango MB, Torres-Carvajal O. 2020. Thermal biology of two tropical lizards from the Ecuadorian Andes and their vulnerability to climate change. PLoS One 15:e0228043. 10.1371/journal.pone.0228043 31978205 PMC6980609

[bib21] Gunderson AR, Leal M. 2012. Geographic variation in vulnerability to climate warming in a tropical Caribbean lizard. Funct Ecol 26:783–93. 10.1111/j.1365-2435.2012.01987

[bib22] Gunderson AR, Mahler DL, Leal M. 2018. Thermal niche evolution across replicated Anolis lizard adaptive radiations. Proc. R. Soc. B. Biol. Sci. 285:20172241. 10.1093/10.1098/rspb.2017.2241 PMC5936720 29669895

[bib23] Gunderson AR, Stillman JH. 2015. Plasticity in thermal tolerance has limited potential to buffer ectotherms from global warming. Proc R Soc B: Biol Sci 282:20150401. 10.1098/rspb.2015.0401 PMC4455808 25994676

[bib24] Herrel A, James RS, Van Damme R. 2007. Fight versus flight: physiological basis for temperature-dependent behavioral shifts in lizards. J Exp Biol 210:1762–7. 10.1242/jeb.003426 17488939

[bib25] Hertz PE, Huey RB, Stevenson RD. 1993. Evaluating temperature regulation by field-active ectotherms: the fallacy of the inappropriate question. Am Nat 142:796–818. 10.1086/285573 19425957

[bib26] Huey RB, Kearney MR, Krockenberger A, Holtum JAM, Jess M, Williams SE. 2012. Predicting organismal vulnerability to climate warming: roles of behaviour, physiology and adaptation. Philos Trans R Soc B: Biol Sci 367:1665–79. 10.1098/rstb.2012.0005 PMC3350654 22566674

[bib27] Huey R, Slatkin M. 1976. Cost and benefits of lizard thermoregulation. Q Rev Biol 51:363–84. 10.1086/409470 981504

[bib28] Huey RB, Stevenson RD. 1979. Integrating thermal physiology and ecology of ectotherms: a discussion of approaches. Am Zool 19:357–66. 10.1093/icb/19.1.357

[bib5a] India Meteorological Department . 2025. Climate of Karnataka (Climatological Summaries of States Series No. 28, Report No. ESSO/MoES/IMD/CRS/CLI-REP(KAR)/01(2025)/20). Office of Climate Research and Services, IMD, Pune. https://imdpune.gov.in/library/public/Climate%20of%20Karnataka.pdf

[bib29] IPCC 2021. Summary for policymakers. In Climate Change 2021: The Physical Science Basis. Contribution of Working Group I to the Sixth Assessment Report of the Intergovernmental Panel on Climate Change (eds Masson-Delmotte V., Zhai P., Pirani A., Connors S. L., Péan C., Berger S., Caud N., Chen Y., Goldfarb L., Gomis M. I., Huang M., Leitzell K., Lonnoy E., Matthews J. B. R., Maycock T. K., Waterfield T., Yelekçi O., Yu R., Zhou B.). Cambridge: Cambridge University Press. 10.1017/9781009157896

[bib30] Kingsolver JG, Diamond SE, Buckley LB. 2013. Heat stress and the fitness consequences of climate change for terrestrial ectotherms. Funct Ecol 27:1415–23. 10.1111/1365-2435.12145

[bib31] Kubisch EL, Fernández JB, Ibargüengoytía NR. 2011. Is locomotor performance optimised at preferred body temperature? A study of *Liolaemus pictus argentinus* from northern Patagonia, Argentina. J Therm Biol 36:328–33. 10.1016/j.jtherbio.2011.06.006

[bib32] Labra A, Soto-Gamboa M, Bozinovic F. 2001. Behavioral and physiological thermoregulation of Atacama desert-dwelling Liolaemus lizards. Ecoscience 8:413–20. https://www.jstor.org/stable/42901355

[bib33] Lima FP, Gomes F, Seabra R, Wethey DS, Seabra MI, Cruz T, Santos AM, Hilbish TJ. 2016. Loss of thermal refugia near equatorial range limits. Glob Change Biol 22:254–63. 10.1111/gcb.13115 26426985

[bib34] Logan ML, Cox CL. 2020. Genetic constraints, transcriptome plasticity, and the evolutionary response to climate change. Front Genet 11:538226. 10.3389/fgene.2020.538226 33193610 PMC7531272

[bib35] Logan ML, Huynh RK, Precious RA, Calsbeek RG. 2013. The impact of climate change measured at relevant spatial scales: new hope for tropical lizards. Glob Change Biol 19:3093–102. 10.1111/gcb.12253 23661358

[bib36] Martin TL, Huey RB. 2008. Why “suboptimal” is optimal: jensen’s inequality and ectotherm thermal preferences. Am Nat 171:E102–18. 10.1086/527502 18271721

[bib37] Medina M, Fernández JB, Charruau P, de la Cruz FM, Ibargüengoytía N. 2016. Vulnerability to climate change of *Anolis allisoni* in the mangrove habitats of Banco Chinchorro Islands, Mexico. J Therm Biol 58:8–14. 10.1016/j.jtherbio.2016.02.005 27157328

[bib38] Neel LK, Logan ML, Nicholson DJ, Miller C, Chung AK, Maayan I, Degon Z, DuBois M, Curlis JD, Taylor Q et al. 2021. Habitat structure mediates vulnerability to climate change through its effects on thermoregulatory behavior. Biotropica 53:1121–33. 10.1111/btp.12951

[bib39] Parmesan C, Yohe G. 2003. A globally coherent fingerprint of climate change impacts across natural systems. Nature 421:37–42. 10.1038/nature01286 12511946

[bib40] Peig J, Green AJ. 2009. New perspectives for estimating body condition from mass/length data: the scaled mass index as an alternative method. Oikos 118:1883–91. 10.1111/j.1600-0706.2009.17643.x

[bib41] Pincebourde S, Murdock CC, Vickers M, Sears MW. 2016. Fine-scale microclimatic variation can shape the responses of organisms to global change in both natural and urban environments. Integr Comp Biol 56:45–61. 10.1093/icb/icw016 27107292

[bib7a] Pontes-da-Silva E, Magnusson WE, Sinervo B, Caetano GH, Miles DB, Colli GR, Diele-Viegas LM, Fenker J, Santos JC, Werneck FP. 2018. Extinction risks forced by climatic change and intraspecific variation in the thermal physiology of a tropical lizard. J Therm Biol 73:50–60. 10.1016/j.jtherbio.2018.01.013 29549991

[bib8a] R Core Team . 2024. R: A Language and Environment for Statistical Computing. Vienna, Austria: R Foundation for Statistical Computing. https://www.R-project.org/

[bib43] Radder RS, Saidapur SK, Shanbhag BA. 2005. Population density, microhabitat use and activity pattern of the Indian rock lizard, *psammophilus dorsalis* (Agamidae). Curr Sci 89:560–6.

[bib44] Rubalcaba JG, Olalla-Tárraga MÁ. 2020. The biogeography of thermal risk for terrestrial ectotherms: scaling of thermal tolerance with body size and latitude. J Anim Ecol 89:1277–85. 10.1111/1365-2656.13181 31990044

[bib45] Sinervo B, Méndez-de-la-Cruz F, Miles DB, Heulin B, Bastiaans E, Villagrán-Santa Cruz M, Lara-Resendiz R, Martínez-Méndez N, Calderón-Espinosa ML, Meza-Lázaro RN et al. 2010. Erosion of lizard diversity by climate change and altered thermal niches. Science 328:894–9. 10.1126/science.1184695 20466932

[bib46] Slatyer RA, Hirst M, Sexton JP. 2013. Niche breadth predicts geographical range size: a general ecological pattern. Ecol Lett 16:1104–14. 10.1111/ele.12140 23773417

[bib47] Stevenson RD . 1985. The relative importance of behavioral and physiological adjustments controlling body temperature in terrestrial ectotherms. Am Nat 126:362–86. https://doi.org/https://www.jstor.org/stable/2461361

[bib48] Sunday JM, Bates AE, Dulvy NK. 2011. Global analysis of thermal tolerance and latitude in ectotherms. Proc R Soc B: Biol Sci 278:1823–30. 10.1098/rspb.2010.1295 PMC3097822 21106582

[bib49] Sunday JM, Bates AE, Kearney MR, Colwell RK, Dulvy NK, Longino JT, Huey RB. 2014. Thermal-safety margins and the necessity of thermoregulatory behavior across latitude and elevation. Proc Natl Acad Sci 111:5610–5. 10.1073/pnas.1316145111 24616528 PMC3992687

[bib50] Tatu A, Dutta S, Thaker M. 2024. Hotter deserts and the impending challenges for the spiny-tailed lizard in India. Biol Open 13:bio060150. 10.1242/bio.060150 38466074 PMC11007731

[bib51] Tewksbury JJ, Huey RB, Deutsch CA. 2008. Putting the heat on tropical animals. Science 320:1296–7. 10.1126/science.1159328 18535231

[bib52] Valenzuela-Ceballos S, Castañeda G, Rioja-Paradela T, Carrillo-Reyes A, Bastiaans E. 2015. Variation in the thermal ecology of an endemic iguana from Mexico reduces its vulnerability to global warming. J Therm Biol 48:56–64. 10.1016/j.jtherbio.2014.12.011 25660631

[bib53] Vivas GL, Robles CI, Halloy M. 2019. Substrate use and its effect on body temperature in two syntopic Liolaemus lizards in northwestern Argentina. Basic Appl Herpetol 33:69–80. 10.11160/bah.160.

[bib54] Wood SN . 2015. Package 'mgcv'. R package version 1.9-3. Available at: 10.1093/10.32614/CRAN.package.mgcv (Accessed 28 July 2026).

