# Supplementary material for: Co-occurring Agamid Lizards Vary in Thermal Vulnerability Despite Similar Climatic Exposure
Source: Integr Org Biol. 2026 Jul 24;8(1):obag041. doi: 10.1093/iob/obag041 (PMC7619449; doi:10.1093/iob/obag041)
Supplement: Supplementary file 1 [file obag041_supplemental_files.zip › Ben_iob_SI_1_revision_May25.docx]

#### **Supplementary information 1**

a. ***Laboratory calibration of physical model temperatures and live lizards body temperature***

Methods: Wild adult males of *C. versicolor* and *P. dorsalis* (n = 3 each per species) were caught and housed in the lizard room at CES. To determine the degree to which copper models (Te) correlate with cloacal temperature, we placed one adult sized copper model with Thermochron iButton (Maxim Integrated, DS1921G) placed inside and one lizard in a metal container immersed in a water bath. Cloacal temperature (Tb) was recorded using a Type-K thermocouple (Amprobe TMD-50), inserted 10 mm into the cloaca and secured to the base of the tail with surgical tape. All trials began with lizards at an initial body temperature of ~27 °C, and cloacal temperatures and copper model temperatures were recorded every minute. The water bath temperature was increased to raise Tb at ~1 °C per minute until it reached the CTmax range of respective lizard species. Similarly, using ice cubes, lizard Tb was reduced at a rate of~1 °C per minute until it reached the CTmin range. A 24hr gap was given between the trials. For each species, we fitted linear mixed-effects models with Tb as the response variable and iButton temperature as the predictor, including individual identity as a random intercept to account for repeated measurements. Both linear and quadratic models were evaluated, and model selection was based on likelihood ratio tests under maximum likelihood estimation

Results: Copper model temperatures were strongly correlated with lizard body temperatures in both species. In *P. dorsalis*, copper model temperature explained 97.3% of the variation in body temperature (marginal R² = 0.973), and the relationship was significant (likelihood ratio test: χ² = 797.71, df = 2, P < 0.001). Similarly, in *C. versicolor*, copper model temperature explained 98.0% of the variation in body temperature (marginal R² = 0.980; χ² = 949.98, df = 2, P < 0.001). (Figure S1a). Species-specific quadratic calibration equations describing the relationship between copper model temperature (iButton) and cloacal body temperature (Tb) were:

*P. dorsalis,* Tb= 3.901+ 1.466*iButton -0.014* iButton^2^

*C. versicolor,* Tb= 10.658+ 0.938 * iButton- -0.004 * iButton^2^

These equations were subsequently used to calibrate field-recorded operative temperatures.

**Figure S1a**: Calibration of copper model temperature. Copper model temperatures (iButton) are plotted against cloacal body temperatures (Tb) for (A) *C. versicolor* (blue) and (B) *P. dorsalis* (pink). Open circles represent observed paired measurements for individual lizards. The dashed line indicates the 1:1 relationship, where copper model temperature equals body temperature. The solid red line shows the species-specific quadratic calibration curve derived from mixed-effects models. These curves were used to correct copper model temperatures to accurately predict lizard body temperature across the full thermal range.

b. ***Laboratory calibration of physical models and live lizards body temperature with environmental temperature***

To evaluate the performance of physical models relative to live lizards at different environmental temperatures, we conducted laboratory calibration experiments in a stainless-steel container immersed in a water bath. For each trial, we simultaneously monitored:

1. One physical model of lizard (made using 31-gauge (0.295 mm) copper sheets as hollow cylinders with closed ends that mimic the dimensions of a typical adult lizard of both species -12 cm length and 9 cm maximum circumference, containing a Thermochron iButton (DS1921G) inside.

2. One live adult lizard with a calibrated Type-K thermocouple probe inserted ~10 mm into the cloaca to measure core body temperature.

3. Two “naked” iButtons (exposed directly to container) to record ambient environmental temperature (average value of both ibuttons were used in calculations and referred as environmental temperature).

Procedure: Trials were conducted for both *P. dorsalis* (n=3) and *C. versicolor* (n=3). Trials began at an initial water temperature of 15–17°C. Physical model, lizard and ibuttons were placed in a metal container immersed in water bath. Lizards and models were allowed to acclimatize for 30 minutes. Water temperature was then increased in a stepwise manner by approximately 2°C every 10 minutes. Temperatures were recorded continuously every 5 minutes until the upper thermal limit of lizard was reached. Linear regressions were used to examine relationships between environmental temperature and temperatures of live lizards (Tb) and physical models. For each trial, we fitted two models: lizard body temperature ~ environmental temperature and physical model temperature ~ environmental temperature. Analyses were performed in R using ordinary least squares regression. Model fit was assessed using slope, intercept, R², and RMSE.

Results: Linear relationships were observed between environmental temperature and both lizard body temperature (slopes 0.530–0.745, R² 0.880–0.935) and physical model temperature (slopes 0.596–0.800, R² 0.903–0.987) across both *P. dorsalis* and *C. versicolor*. Although physical models generally showed slightly steeper slopes and higher R² values than lizards, calibration plots consistently showed that models recorded lower temperatures than live lizards during the heating phase in the ecologically relevant range (~28–42 °C) (Figure S1b). This indicates that the physical models have greater thermal inertia than the live lizards. High overall correlations (Supplementary information 1A) confirm that the models track ambient conditions well, but they do not equilibrate as rapidly as the study species. In the field, temperatures recorded by these models should therefore be interpreted as a proxy for microenvironmental temperatures rather than strictly as operative temperatures (Tₑ) following Bakken (1992).

**Figure S1b**: Relationship between environment temperature and lizard body/physical model temperature during laboratory calibration trials. Physical model temperatures (blue) and lizard body temperature (red) are plotted against environmental temperature for (A) *C. versicolor* and (B) *P. dorsalis*. The dashed line represents the 1:1 relationship.
