# Supplementary material for: Co-occurring Agamid Lizards Vary in Thermal Vulnerability Despite Similar Climatic Exposure
Source: Integr Org Biol. 2026 Jul 24;8(1):obag041. doi: 10.1093/iob/obag041 (PMC7619449; doi:10.1093/iob/obag041)
Supplement: Supplementary file 1 [file obag041_supplemental_files.zip › Ben_iob_SI_2_revision_May25.docx]

#### **Supplementary information 2**

**Figure S2a**

**Figure S2a**: Interaction effect of species and microhabitat on physical model temperature. Point estimates represent the estimated marginal means derived from the linear model, while vertical bars indicate 95% confidence intervals. The plot illustrates the thermal environments experienced by *C. versicolo*r (blue) and *P. dorsalis* (pink) in open and shaded microhabitats.

**Figure S2b**

**Figure S2b**: Hourly mean microhabitat temperature measured using physical models. Line plots illustrate the hourly thermal variation measured during the peak summer month (May) for microhabitats occupied by *C. versicolor* and *P. dorsalis*. Colors distinguish specific microhabitats across open and shaded conditions. Note the extreme thermal peaks observed in exposed substrates around midday, contrasting with the relative stability of shaded refugia.
